# YOLO11s-UAV: An Advanced Algorithm for Small Object Detection in UAV Aerial Imagery

**DOI:** 10.3390/jimaging12020069

**Published:** 2026-02-06

**Authors:** Qi Mi, Jianshu Chao, Anqi Chen, Kaiyuan Zhang, Jiahua Lai

**Affiliations:** 1 College of Mechanical and Electrical Engineering, Fujian Agriculture and Forestry University, Fuzhou 350108, China; 52312047025@fafu.edu.cn (Q.M.); 52412047001@fafu.edu.cn (A.C.); 2 Quanzhou Institute of Equipment Manufacturing, Haixi Institutes, Chinese Academy of Sciences, Quanzhou 362216, China; 238527305@fzu.edu.cn (K.Z.); laijiahua@fjirsm.ac.cn (J.L.); 3 Fujian Institute of Research on the Structure of Matter, Chinese Academy of Sciences, Fuzhou 350025, China; 4 Fujian College, University of Chinese Academy of Sciences, Fuzhou 350025, China; 5 School of Advanced Manufacturing, Fuzhou University, Quanzhou 362251, China

**Keywords:** UAV aerial imagery, small object detection, feature pyramid, lightweight object detector, YOLO11s, VisDrone-DET2019

## Abstract

Unmanned aerial vehicles (UAVs) are now widely used in various applications, including agriculture, urban traffic management, and search and rescue operations. However, several challenges arise, including the small size of objects occupying only a sparse number of pixels in images, complex backgrounds in aerial footage, and limited computational resources onboard. To address these issues, this paper proposes an improved UAV-based small object detection algorithm, YOLO11s-UAV, specifically designed for aerial imagery. Firstly, we introduce a novel FPN, called Content-Aware Reassembly and Interaction Feature Pyramid Network (CARIFPN), which significantly enhances small object feature detection while reducing redundant network structures. Secondly, we apply a new downsampling convolution for small object feature extraction, called Space-to-Depth for Dilation-wise Residual Convolution (S2DResConv), in the model’s backbone. This module effectively eliminates information loss caused by strided convolution or pooling operations and facilitates the capture of multi-scale context. Finally, we integrate a simple, parameter-free attention module (SimAM) with C3k2 to form Flexible SimAM (FlexSimAM), which is applied throughout the entire model. This improved module not only reduces the model’s complexity but also enables efficient enhancement of small object features in complex scenarios. Experimental results demonstrate that on the VisDrone-DET2019 dataset, our model improves mAP@0.5 by 7.8% on the validation set (reaching 46.0%) and by 5.9% on the test set (increasing to 37.3%) compared to the baseline YOLO11s, while reducing model parameters by 55.3%. Similarly, it achieves a 7.2% improvement on the TinyPerson dataset and a 3.0% increase on UAVDT-DET. Deployment on the NVIDIA Jetson Orin NX SUPER platform shows that our model achieves 33 FPS, which is 21.4% lower than YOLO11s, confirming its feasibility for real-time onboard UAV applications.

## 1. Introduction

With the continuous advancement of technology, the production costs of unmanned aerial vehicles (UAVs) have significantly decreased, leading to their widespread adoption in various applications [1,2]. For example, in precision agriculture [3], UAVs employ Artificial Intelligence algorithms to process sensor data for vegetation monitoring and analysis, such as mapping and detecting highly invasive weeds within heterogeneous terrains of the North American prairie ecosystems. In urban traffic management [4], UAVs deliver high-resolution imagery for topographic mapping, infrastructure monitoring, traffic analysis, and environmental assessment, supporting data-driven urban planning. In search and rescue operations [5], UAVs enhance efficiency through rapid damage assessment, victim location, payload delivery, and data relay, enabling access to inaccessible areas and timely response. Consequently, vision-based object detection from UAV platforms has emerged as a key research topic, particularly for recognizing small objects in aerial imagery under real-world sensing conditions. The challenges associated with UAV small object detection primarily stem from several key factors [6,7]. First, the high-altitude operation of UAVs results in objects occupying only a small number of pixels in the captured images, making accurate detection and recognition difficult. Second, the deployment environment often features complex and diverse backgrounds, such as dense urban areas or natural landscapes, accompanied by other challenging conditions like overexposure, low illumination, and occlusion of small objects, which can introduce significant background interference and visual confusion, thereby reducing the distinguishability of objects. Third, the limited onboard resources constrain the model’s size and complexity, making it challenging to deploy large-scale deep learning models that require substantial memory.

For general object detection, current research has primarily focused on improved feature pyramid networks capable of handling multi-scale feature fusion, more efficient convolution modules for enhancing image feature downsampling, attention mechanisms that guide models to dynamically focus on key regions while suppressing irrelevant complex background interference, and other techniques. However, these approaches still have limitations in meeting the specific demands of UAV-based small object detection.

Regarding multi-scale feature fusion, the pioneering work of the Feature Pyramid Network (FPN) [8], introduced in 2017, establishes a top-down architecture with lateral connections to effectively fuse multi-scale features. Building upon this foundation, subsequent variants have sought to enhance its capabilities. For instance, PAFPN [9] further augments the architecture with additional bottom-up paths, while NAS-FPN [10] employs neural architecture search to discover optimized pyramid connections. However, these enhanced variants suffer from inefficient computational cost and excessive parameters, which are prohibitive for resource-constrained UAV platforms. Moreover, aggressive downsampling in their backbone networks severely degrades spatial details, critically undermining small object detection confidence.

In the pursuit of efficient downsampling, DSConv [11] decomposes standard convolution into depthwise and pointwise convolutions, effectively mitigating information loss during traditional downsampling while constructing lighter and deeper networks. ODConv [12] further introduces a multi-dimensional attention mechanism, enabling dynamic adjustment of convolution kernel parameters. GhostConv [13] generates redundant feature maps through inexpensive linear transformations, preserving more feature information at an extremely low computational cost and offering potential for lightweight model deployment. Although these advancements in downsampling convolutions have significantly improved accuracy in general object detection and effectively reduced model complexity, they share a common weakness for small objects because they often lose crucial spatial details of small objects during strided convolution or pooling operations.

Concurrently, while Softmax Attention in Transformers [14] can efficiently capture global information, its O(N^2^) complexity results in high computational costs when processing a large number of visual tokens. Although Linear Attention [15] reduces the complexity to linear O(N), it neglects the Query magnitude information. MALA [16] addresses this by incorporating Query magnitude into Linear Attention while maintaining linear complexity, achieving a balance between efficiency and performance, yet it still imposes substantial computational overhead compared to lightweight attention mechanisms. Furthermore, Bi-level Routing Attention, as proposed in BiFormer [17], enables the simultaneous capture of both global and local feature information by introducing a dual-level routing mechanism, thereby enhancing model performance on vision tasks. However, this improvement leads to higher computational demands and extended training durations, hindering its deployment on UAV platforms with limited resources and strict latency requirements.

These shortcomings above motivate us to develop a more efficient and targeted solution. To bridge this gap, we propose YOLO11s-UAV, an enhanced version of YOLO11s specifically designed to maintain high detection performance for small objects in aerial imagery while being amenable to resource-constrained UAV platforms. Our model not only significantly improves the detection performance for small objects in complex aerial environments but also adopts a lightweight architecture with considerably fewer parameters, resulting in lower memory overhead and better compatibility with the constrained storage and processing capabilities of UAV systems. Our contributions are summarized as follows:

- Firstly, we propose a novel Content-Aware Reassembly and Interaction Feature Pyramid Network (CARIFPN). This structure integrates the efficient and lightweight CARAFE upsampling operator. It also utilizes skip connections to support feature fusion across different scales. Simultaneously, it eliminates redundant downsampling operations throughout the network. This approach not only reduces unnecessary network structures but also significantly improves the ability to capture features of small objects.
- Secondly, we design a new downsampling convolution module called Space-to-Depth for Dilation-wise Residual Convolution (S2DResConv) for small object feature extraction. This module is applied in the backbone of the model. It effectively prevents the information loss caused by strided convolution or pooling operations. Additionally, it facilitates the capture of multi-scale contextual information.
- Finally, we combine the lightweight attention mechanism SimAM with the C3k2 module to form Flexible SimAM (FlexSimAM). This enhanced module is applied throughout the entire model. It not only reduces the model’s complexity but also enhances its focus on small object features in complex environments. Furthermore, a Boolean configuration is introduced into the C3k2 module. This allows flexible switching between FlexSimAM and the standard C2f structure, significantly increasing the flexibility of its application in different scenarios.

In this paper, we validate the feasibility and effectiveness of module stacking through ablation experiments on the VisDrone-DET2019 [18] dataset. We also perform extensive comparative experiments with other two-stage and one-stage models. Additionally, we conduct generalization experiments on other UAV-based datasets, including TinyPerson [19] and UAVDT-DET [20]. Furthermore, visualization experiments are conducted to intuitively illustrate the results, followed by hardware performance tests on the NVIDIA Jetson Orin NX SUPER platform to evaluate practical deployment potential and validate the model’s real-time capability. Our code is available at https://github.com/Mickey2610/YOLO11s-UAV.git (accessed on 16 January 2026).

## 2. Related Work

### 2.1. Commonly Used Object Detection Models

Currently, there are many general-purpose object detection models, which can be broadly categorized into two types. The first type consists of two-stage models, which are capable of handling complex detection tasks but tend to be slower and are often unsuitable for real-time applications. Notable examples include Faster Region-based Convolutional Neural Network (Faster R-CNN) [21] and Cascade R-CNN [22]. Faster R-CNN uses a Region Proposal Network (RPN) to share convolutional features, speeding up the detection process. Cascade R-CNN improves performance through a multi-stage network and incorporates dynamic pruning and quantization to reduce model size and computational cost.

The second category consists of one-stage models, which are known for their fast detection speeds. This characteristic makes them well-suited for real-time applications. These models are generally simpler and easier to deploy. For example, RetinaNet [23] achieves end-to-end detection using a single network. It addresses class imbalance through the use of Focal Loss. The Feature-Selective Anchor-Free module (FSAF) [24] improves upon the Feature Pyramid Network (FPN) by employing a more strongly supervised detection head. This head assigns targets to the most suitable feature branch. Among one-stage models, the You Only Look Once (YOLO) series is the most widely recognized. YOLOv5 [25] builds on the design philosophy of YOLOv3 [26]. Its early versions underperformed compared to YOLOv3–Spatial Pyramid Pooling (SPP). However, subsequent releases such as YOLOv5 r5.0 and r7.0 improved speed–precision trade-offs and enhanced detection of small, densely packed objects. YOLOv8 [27] uses an enhanced Cross-Stage Partial Darknet (CSPDarknet) backbone. It replaces the C3 module with the more efficient C2f (channel-to-feature) module and adds the Spatial Pyramid Pooling Fast (SPPF) module to handle varying output sizes. For feature fusion, YOLOv8 employs Path Aggregation Network—Feature Pyramid Network (PAN-FPN), which integrates multi-scale features via upsampling and C2f modules. YOLOv9 [28] introduces programmable gradient information (PGI) to improve gradient flow and data retention. It also extends the Efficient Layer Aggregation Network (ELAN) architecture to Generalized-ELAN (G-ELAN), boosting both accuracy and inference speed. YOLOv10 [29] shares similarities with YOLOv6 3.0 [30]. It incorporates Transformer-based modules for global feature extraction and end-to-end detection. The design integrates the Cross-Stage Partial Network (CSPNet) and self-attention mechanisms, resembling the Detection Transformer (DETR) [31] approach. YOLOv12 [32] introduces an attention-centric design along with the novel A2C2f module. This enhances feature aggregation and computational efficiency, outperforming earlier Convolutional Neural Network (CNN)-based YOLO frameworks. YOLOv13 [33] proposes a Hypergraph-based Adaptive Correlation Enhancement (HyperACE) mechanism. It overcomes limitations in local pairwise correlation modeling by capturing global, multi-to-multi high-order dependencies through hypergraph computation.

### 2.2. Recent Studies Related to UAVs

In recent years, numerous studies have focused on developing specialized small object detection algorithms for aerial images captured by UAVs. These algorithmic advances aim to enhance detection performance through optimized model architectures, improved feature fusion strategies, innovative loss functions, and other enhancements, while ensuring that the models remain lightweight and suitable for UAV memory constraints.

Many YOLO-based models have been introduced to advance small object detection in UAV imagery. For instance, Zhao et al. [34] developed MS-YOLOv7. This model integrates the Convolutional Block Attention Module (CBAM) and a novel pyramid pooling module called SPPFS. These additions optimize multi-scale feature extraction and dense object detection. Tahir et al. [35] proposed PVswin-YOLOv8s. It incorporates a Swin Transformer block into the YOLOv8s backbone. This enhances global feature extraction. The model also uses Soft Non-Maximum Suppression (Soft-NMS) to improve detection of pedestrians and vehicles under occlusion. Li et al. [36] introduced SOD-YOLO. This approach integrates the Receptive Field Convolutional Block Attention Module (RFCBAM) into the backbone network. It also presents the Balanced Spatial and Semantic Information Fusion Pyramid Network (BSSI-FPN). These innovations improve feature representation and multi-scale information fusion. Wang et al. [37] developed CPDD-YOLOv8. The model integrates a Global Attention Mechanism into the C2f structure. It adds a P2 detection layer (a high-resolution feature layer) to better capture shallow features. Additionally, it employs Dynamic Snake Convolution in the bottleneck and uses a Dynamic Head with multi-attention mechanisms. These enhancements collectively improve small object detection. Liu et al. [38] presented the DeNoising feature pyramid network with Transformer R-CNN (DNTR). This method combines a customized DeNoising-FPN with a Transformer-based detection head. The aim is to improve detection performance in complex scenes. Ma et al. [39] proposed a model that includes a Scale Decoupling Module, Sparse Nonlocal Attention, and an Adaptive Anchor Matching Strategy. These components work together to enhance the representation and detection of small objects. Wu et al. [40] introduced RC-SODet. The model embeds Reparameterized Dual Convolutions (RepDuConvs) in the backbone. It also introduces the Compact Feature-enhanced Pyramid Network (RC-FPN) as the neck. This design improves inference efficiency through branch fusion and strengthens multi-scale feature representation.

## 3. Methods

### 3.1. Architecture of YOLO11

YOLO11 [41], building on the foundation of YOLOv8, adopts an improved backbone and neck architecture to enhance feature extraction capabilities, increasing detection accuracy and speed in complex tasks while yielding a more lightweight model. Specifically, compared to the YOLOv8 model, YOLO11 replaces the C2f block with C3k2, where the use of either the bottleneck or C3 module is determined by the Boolean state of the c3k parameter. Furthermore, a C2PSA module is added after the Spatial Pyramid Pooling—Fast (SPPF) [25] module. C2PSA extends the C2f module by incorporating a Pointwise Spatial Attention (PSA) [29] block, which enhances feature extraction and attention mechanisms. By integrating the PSA block into the standard C2f module, C2PSA implements a more powerful attention mechanism, improving the model’s ability to capture important features. Additionally, the head design from YOLOv10 is incorporated into YOLO11, utilizing Depthwise Separable Convolution (DSConv) [11] to reduce redundant computations and improve efficiency. Table 1 shows the performance of five different scales of the YOLO11 model trained on the VisDrone-DET2019 dataset. After balancing accuracy and model complexity, we select YOLO11s as our baseline algorithm.

### 3.2. Architecture of Improved Model YOLO11s-UAV

**Figure 1:**
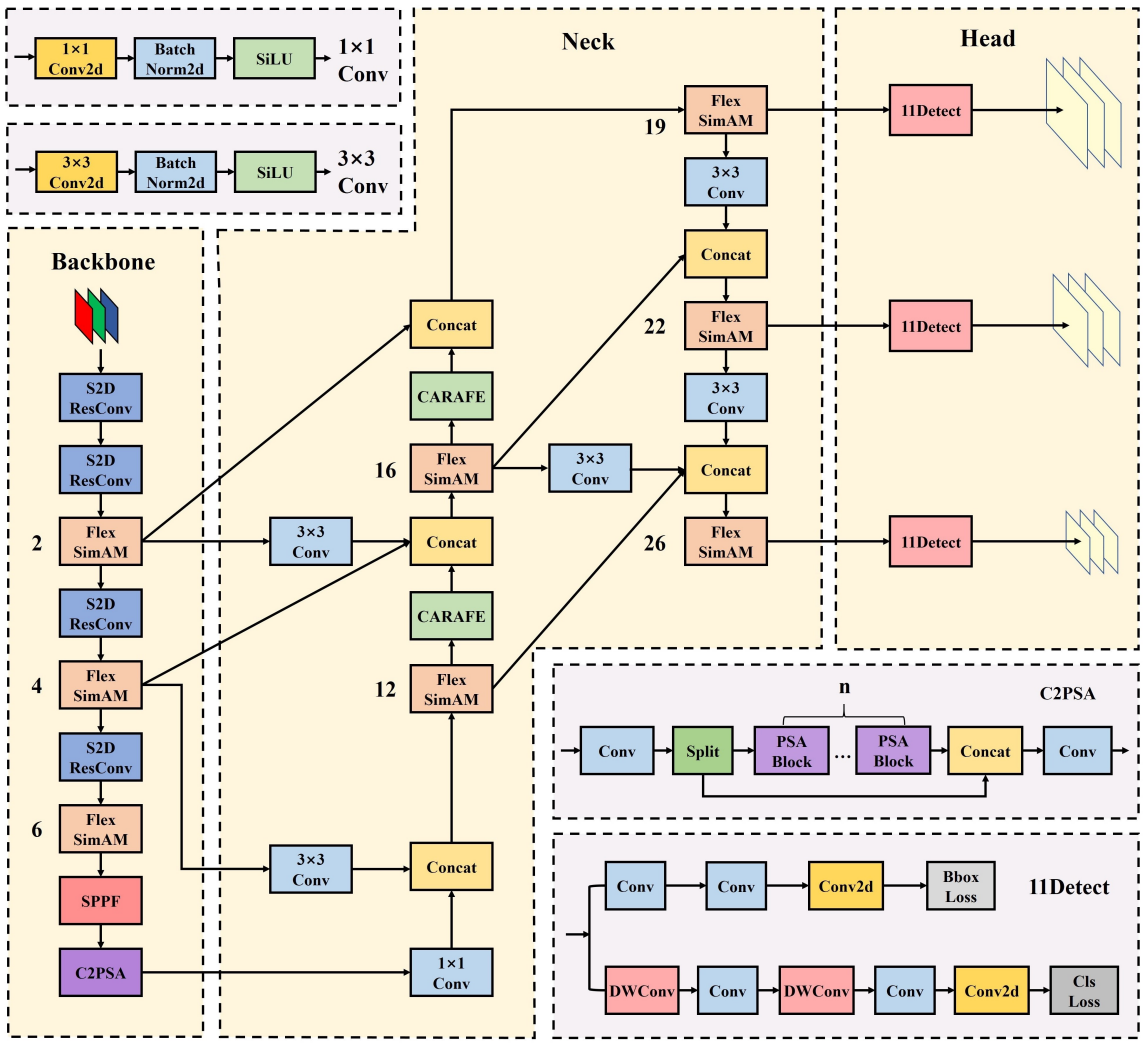
Diagram of the improved model YOLO11s-UAV, which consists of three components: first, the backbone includes four downsampling layers; second, the neck incorporates skip-layer connections with two upsampling operations; and third, the head outputs three feature maps at different levels. The numbers (e.g., 2, 4, 6) denote the current network depth (layer count) where the FlexSimAM module is applied.

Although YOLO11 has achieved lightweight improvements that significantly reduce model complexity and deliver better mAP performance on the MS COCO [42] dataset, it still struggles to extract features from small objects in low-resolution images and complex UAV environments. To address this issue, we propose a lightweight YOLO11s-UAV model specifically optimized for small object detection in UAV scenarios. Our improvements include three key modifications: revising the existing Feature Pyramid Network (FPN), integrating an attention mechanism into the C3k2 module, and designing a dedicated downsampling convolution module for small object feature extraction. The overall structure of the improved model is illustrated in Figure 1.

### 3.3. CARIFPN Structure

In object detection, Feature Pyramid Networks (FPNs) [8] and their successors serve as multi-scale feature representation methods designed to detect objects of varying sizes. For instance, PAFPN [9], widely used in the YOLO series, is primarily developed for general object detection and aims to cover objects across large, medium, and small scales. However, such designs often incorporate excessive downsampling in the backbone network, leading to computational inefficiency and a large number of parameters. Consequently, small object feature information is significantly lost early in the model’s backbone, which prevents these methods from adequately accounting for the detection of even smaller-scale object features captured from a UAV perspective. These limitations restrict their application in resource-constrained scenarios, particularly in UAV-based small object detection. Furthermore, existing multi-scale fusion connections for small object detection are often inefficient, failing to fully achieve complementary enhancement across hierarchical features.

Recent structures like the Efficient-RepGFPN in DAMO-YOLO [43] have made progress in efficiency. For example, compared with Generalized-PFN (GFPN) [44], they remove redundant upsampling operations in the neck, utilize skip-layer connections, and leverage varying channel dimensions across different scales of feature maps. These improvements enhance flexibility in channel dimension adjustment during feature fusion. However, these methods still fail to adequately address the need for enhanced spatial adaptability and contextual awareness in the feature fusion process, especially for small objects. Therefore, significant opportunities remain for further improving feature representation in this area.

**Figure 2:**
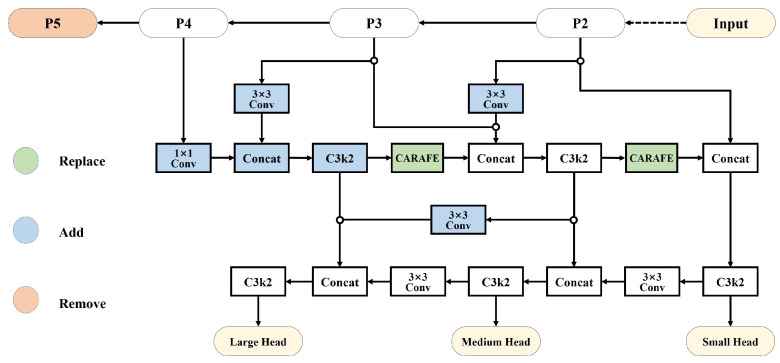
Diagram of CARIFPN. The architecture primarily consists of four downsampling operations in the backbone, along with skip-layer connections and the newly introduced upsampler CARAFE in the neck. Green denotes the replaced components, blue indicates newly added components, and red represents the removed components.

To address the insufficient feature fusion capability in small object detection, inspired by the efficient framework of RepGFPN, we propose a novel Content-Aware Reassembly and Interaction Feature Pyramid Network (CARIFPN), as depicted in Figure 2. Our key improvements include three critical modifications to the original PAFPN. First, we replace the standard upsampling operator with Content-Aware ReAssembly of FEatures (CARAFE) [45]. Second, we reduce the total number of downsampling operations from five to four by removing redundant downsampling layers in the backbone network, which effectively mitigates information loss and preserves crucial spatial details for small object detection. Finally, we introduce skip connections between the backbone and neck networks, consisting of three 3 × 3 convolutions and one channel-adjusting 1 × 1 convolution. This design systematically enhances cross-scale feature fusion capability and semantic information transmission efficiency.

**Figure 3:**
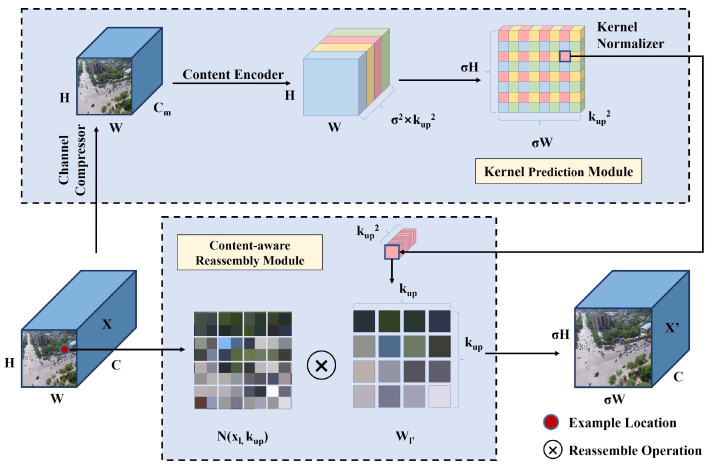
Diagram of CARAFE [45], consisting of Kernel Prediction Module and Content-Aware Reassembly Module.

Within this architecture, CARAFE serves as a lightweight and efficient upsampling module. As illustrated in Figure 3, given an input feature map *X* of size C×H×W and an integer upsampling rate σ, CARAFE produces an upsampled feature map $X^{\prime }$ of size C×σH×σW. Its operation consists of a two-stage process. First, a Kernel Prediction Module ψ predicts a location-specific kernel $W_{l^{\prime }}$ for each location $l^{\prime }$ through channel compression, a content encoder and kernel normalization. This prediction is based on the k×k neighborhood centered at location *l* in the input feature map $X_{l}$, as shown in Equation (1):

(1) $$W_{l^{\prime }}=\psi \left(N\left(X_{l},k_{\mathrm{encoder}}\right)\right),$$

where $k_{\mathrm{encoder}}$ denotes the kernel size of the content encoder in the kernel prediction stage, while $N(X_{l},k)$ denotes the k×k subregion of *X* centered at location *l*.

Subsequently, a Content-Aware Reassembly Module ϕ uses the predicted kernel $W_{l^{\prime }}$ to reassemble features within the local neighborhood of $X_{l}$. By giving more weight to relevant points in the local context, the reassembled feature map can retain richer semantic information compared to the original input. This process yields the output feature map $X_{l^{\prime }}^{\prime }$, as shown in Equation (2):

(2) $$X_{l^{\prime }}^{\prime }=\phi \left(N\left(X_{l},k_{\mathrm{up}}\right),W_{l^{\prime }}\right),$$

where $k_{\mathrm{up}}$ represents the kernel size employed in the feature reassembly stage.

Through these two modules, CARAFE dynamically generates adaptive kernels based on the surrounding context, enabling feature reassembly with an enlarged effective receptive field. This capability is particularly useful for detecting small objects, as it supports the reconstruction of richer and more semantically meaningful feature maps during upsampling.

In summary, CARIFPN is a carefully designed feature pyramid network. It integrates context-aware upsampling, optimized downsampling reduction, and enhanced cross-scale connections. By combining these three core improvements, CARIFPN achieves an improved balance among feature preservation, computational efficiency, and representational capacity. It is specifically tailored for small object detection in UAV-captured aerial imagery. This architecture effectively addresses the limitations of existing FPN variants in resource-constrained scenarios while maintaining strong small object detection performance.

### 3.4. S2DResConv Module

**Figure 4:**
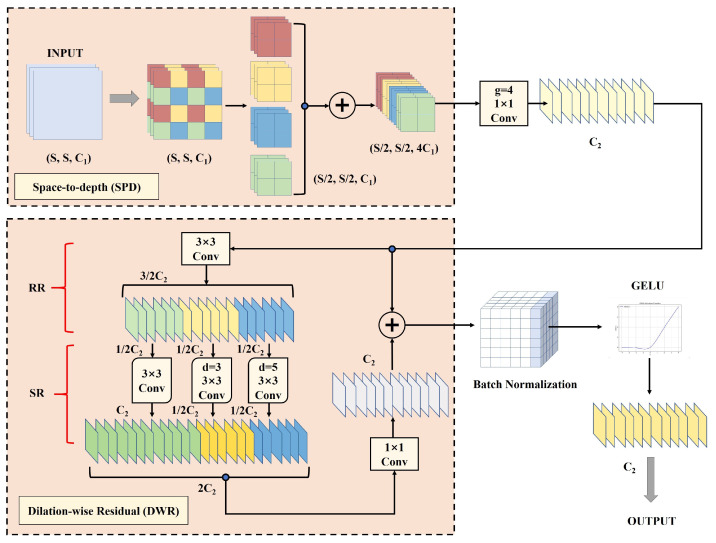
Diagram of S2DResConv, including details of space-to-depth, Dilation-wise Residual and other components.

Building upon the improvements introduced by CARIFPN for enhanced multi-scale feature fusion and representation in UAV-based small object detection, we further address the challenge of feature information loss during downsampling in the backbone network. In recent years, many general-purpose strided convolution modules have been proposed. However, small objects occupy only a limited pixel area in images, and their low resolution often results in insufficient feature information. As a consequence, these standard strided convolution modules tend to lose significant information during downsampling. To address this problem, we propose Space-to-Depth for Dilation-wise Residual Convolution (S2DResConv). This module builds upon the space-to-depth (SPD) technique [46] and the Dilation-wise Residual (DWR) mechanism [47]. Figure 4 illustrates the structure of the S2DResConv module, and the following sections provide a detailed explanation of each step.

The module begins by processing the input feature map through the space-to-depth (SPD) module. Given an input feature map $M\in (S,S,C_{1})$, it is first divided into multiple pixel blocks using a stride of 2. These blocks are then grouped along the spatial dimension according to their positions, forming four distinct sub-blocks: $f_{00},f_{10},f_{01},f_{11}$, as defined in Equation (3):

(3) $$\left\{\begin{array}{cc} & f_{00}=M\left[0:S:2,0:S:2\right]\\ & f_{10}=M\left[1:S:2,0:S:2\right]\\ & f_{01}=M\left[0:S:2,1:S:2\right]\\ & f_{11}=M\left[1:S:2,1:S:2\right]. \end{array}\right.$$

Each resulting sub-block has dimensions $\left(\frac{S}{2},\frac{S}{2},C_{1}\right)$. These sub-blocks then undergo a space-to-depth transformation, which expands the channel dimension to $4C_{1}$ while halving both the height and width. This yields an output feature map of size $\left(\frac{S}{2},\frac{S}{2},4C_{1}\right)$. The SPD module preserves all visual details from the original input, thereby preventing the loss of small object features during the initial downsampling stage.

Next, a Non-stride Convolution with a stride of 1 is applied to the output of the SPD module. This convolution adjusts the channel dimension to the desired size while ensuring compatibility with the input of the following DWR module. Importantly, this step avoids any pooling operations that could lead to feature loss.

The processed features are then fed into the Dilation-wise Residual (DWR) module. Within this module, Regional Residualization (RR) extracts relevant residual features from the input. Meanwhile, Semantic Residualization (SR) applies multi-rate dilated depthwise convolutions to perform morphological filtering on features from regions of varying sizes. After multi-scale contextual information is extracted, a pointwise convolution merges these features to produce the final residual representation. These residuals are added back to the original input feature map, thereby enriching its representation. This multi-scale contextual modeling is essential for distinguishing small objects from complex backgrounds. Moreover, the residual learning framework ensures that the enhanced contextual information supplements, rather than replaces, the fine details preserved in the SPD stage. This results in a more robust and informative feature representation.

Finally, the feature maps undergo batch normalization (BN) and activation function processing. The normalized features are passed through the GELU activation function, which is approximated by Equation (4):

(4) $$\mathrm{GELU}\left(x\right)=\frac{\left[1+\tanh\left(\sqrt{\frac{2}{\pi }}\left(x+0.044715x^{3}\right)\right)\right]}{2}.$$

Applying batch normalization before GELU helps smooth the activation values, leading to more stable outputs and improved gradient flow during training. The GELU activation offers smooth nonlinear transformations, mitigates the dead neuron issue often encountered with ReLU, and enhances the overall expressive capacity of the model.

By integrating the SPD module, non-strided convolution, and the DWR module, we establish a progressive feature enhancement pipeline. This pipeline advances from detail preservation and dimension adjustment to semantic strengthening. The overall design of the module not only effectively prevents the degradation of small object features typically caused by traditional downsampling, but also significantly improves the model’s feature extraction capability, robustness, and accuracy in complex backgrounds. As a result, the backbone network equipped with S2DResConv can better preserve small object features and enhance contextual extraction, allowing more informative representations to be effectively delivered into the neck network through the CARIFPN pathway and ultimately reach the detection head. This enables the final detection outputs to achieve higher sensitivity and precision for small object localization and recognition.

### 3.5. FlexSimAM Module

Based on the enhanced feature fusion pathways of CARIFPN and the improved downsampling representations from S2DResConv, we further optimize the C3k2 module within the CARIFPN architecture. In this structure, the C3k2 module processes intermediate feature maps from preceding layers, especially those produced by S2DResConv in the backbone, to generate new intermediate representations. Through concatenation operations, it enables the transmission of multi-scale features from different backbone stages to the neck network via both baseline connections and the newly introduced skip connections among CARIFPN. It also facilitates cross-branch feature fusion within the neck by integrating information across scales restored through upsampling and further refined through downsampling.

To enhance the sensitivity and representational capacity of this critical module for fine-grained small object features and to align with the limited onboard resources of UAV platforms, we integrate a lightweight attention mechanism, the simple, parameter-free attention module (SimAM) [48], into C3k2 to form Flexible SimAM (FlexSimAM). SimAM introduces 3D attention weights to feature maps without adding parameters, enabling dynamic and efficient enhancement of the network’s representational capacity. This is particularly vital for small object detection, where subtle features are often overwhelmed by complex backgrounds. The attention mechanism selectively amplifies responses from neurons corresponding to small targets while suppressing irrelevant background noise, significantly improving the signal-to-noise ratio in feature representation. Below are detailed descriptions of SimAM.

The complete algorithmic workflow of SimAM is presented in Algorithm 1. Its core lies in efficiently generating an attention mechanism with real 3D weights. To achieve this, it is crucial to evaluate the importance of individual neurons. Drawing on neuroscientific findings such as spatial suppression [49], Yang et al. [48] proposed an energy function to quantify the importance of each neuron. This function is defined as Equation (5):

(5) $$\begin{array}{cc} e_{t}\left(w_{t},b_{t},y,x_{i}\right) & =\frac{1}{M-1}\sum_{i=1}^{M-1}{\left(-1-(w_{t}x_{i}+b_{t})\right)}^{2}\\ & \;+{\left(1-(w_{t}t+b_{t})\right)}^{2}+\lambda w_{t}^{2}, \end{array}$$

where *t* and $x_{i}$ indicate the target neuron and the remaining neurons in a single channel of the input feature map $X\in R^{C\times H\times W}$, respectively. In this function, *i* denotes the spatial dimension and $w_{t}$ and $b_{t}$ correspond to the transformation’s weight and bias. M=H×W represents the total neurons in that channel. The symbol λ denotes a hyperparameter that can be set via cross-validation search.
**Algorithm 1** SimAM module.**function **forward(x)  **b, c, h, w** ← x.size()  n ← w × h − 1  x’ ← (x − x.mean(dim=[2, 3], keepdim=True))^2^  y ←$\frac{x^{\prime }}{4(x^{\prime }.\mathrm{sum}\left(\dim=\left[2,3\right],\mathrm{keepdim}=\mathrm{True}\right)/n+e_{\lambda }}+0.5$  **return** x × sigmoid(y)

To avoid recomputing the mean and variance at each position, the least amount of energy can be efficiently computed as Equation (6):

(6) $$e_{t}^{*}=\frac{4({\hat{\sigma }}^{2}+\lambda )}{{\left(t-\hat{\mu }\right)}^{2}+2{\hat{\sigma }}^{2}+2\lambda },$$

where a lower energy $e_{t}^{*}$ indicates that neuron *t* is more distinctive from its surrounding neurons, making it more relevant for visual processing. Consequently, the importance of each surrounding neuron can be expressed as $1/e_{t}^{*}$. $\hat{\mu }=\frac{1}{M}\sum_{i=1}^{M}x_{i}$ and ${\hat{\sigma }}^{2}=\frac{1}{M}\sum_{i=1}^{M}{\left(x_{i}-\hat{\mu }\right)}^{2}$ denote the estimates of the mean and variance calculated over all neurons within that channel, respectively.

Additionally, attention modulation is typically considered a scaling effect on neuronal responses within mammalian neural systems [50]. To reflect this, a scaling operator is employed to refine the features. The full refinement process of the SimAM module is given in Equation (7):

(7) $$\tilde{X}=\mathrm{sigmoid}\left(\frac{1}{E}⊙X\right),$$

where *E* aggregates all $e_{t}^{*}$ values across both the spatial dimensions and channel, with the sigmoid function applied to prevent excessively large values.

**Figure 5:**
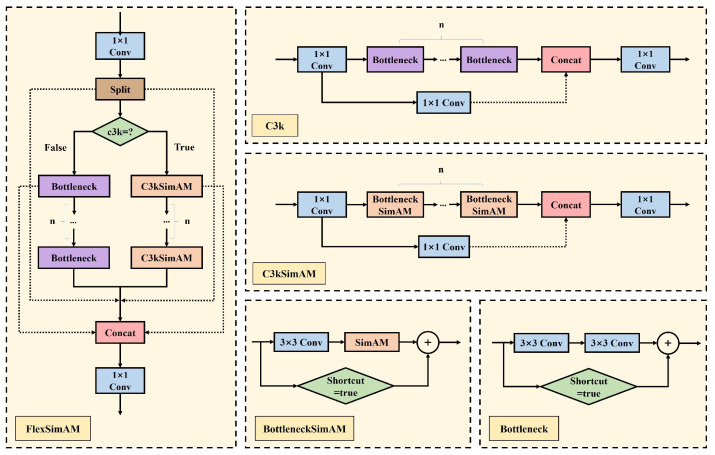
Diagram of FlexSimAM, including details of the C3kSimAM, BottleneckSimAM, and bottleneck, as well as the details of the original C3k. The green diamond-shaped modules, c3k and shortcut, indicate the inclusion of a Boolean value.

The proposed FlexSimAM module, as illustrated in Figure 5, introduces a configurable design as its key innovation. We create the BottleneckSimAM module by replacing the second convolutional layer in the standard bottleneck with the SimAM attention module. Meanwhile, the C3kSimAM structure is formed by replacing the original bottleneck modules in C3k with our proposed BottleneckSimAM module. This hierarchical integration allows dynamic selection between the standard sequential bottleneck and the enhanced C3kSimAM structure through the c3k parameter setting. When the c3k parameter is set to true, the information flows through the attention-enhanced branch containing C3kSimAM. This branch performs deeper feature interaction and extraction. When the parameter is set to false, the information only passes through the bottleneck branch, making its function identical to that of C2f in YOLOv8. Both selectable modes are fused through residual connections and a 1 × 1 convolution at the output. This enables the SimAM attention mechanism to flexibly adapt to the varying representational and computational requirements of different network layers. Building on this flexibility, before model training for UAV scenarios, we can adjust the c3k setting for each FlexSimAM module in both the backbone and neck networks. This lets us decide for each layer whether to use the enhanced C3kSimAM structure or not. By turning on this enhanced structure only where needed, we can better extract small object features in complex UAV environments while keeping the model lightweight, since SimAM adds no extra parameters.

## 4. Experiments

### 4.1. Datasets

**Figure 6:**
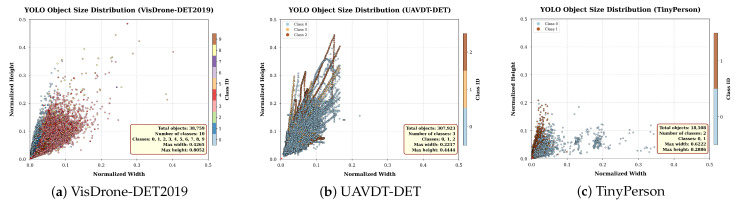
YOLO object size distribution in the validation set of (**a**) VisDrone-DET2019, (**b**) UAVDT-DET, and (**c**) TinyPerson datasets. The object sizes across categories are color-coded. The horizontal axis represents the normalized width, while the vertical axis represents the normalized height.

In this paper, we use publicly available UAV-based datasets, with the VisDrone-DET2019 [18] dataset serving as the main dataset. Additionally, the UAVDT-DET [20] dataset is used to supplement road traffic scenarios, and the TinyPerson [19] dataset is employed to enhance the model’s generalization capability in detecting tiny-scale pedestrians. Figure 6 illustrates the size distribution of manually labeled objects in the validation sets of the three datasets. It shows that all three datasets contain a large number of small objects. Among them, VisDrone-DET2019 exhibits a relatively uniform size distribution, while UAVDT-DET displays a significant variation in object sizes. In contrast, TinyPerson is predominantly concentrated in the extremely small size range. These size distributions reflect the varying altitudes from which the drone-captured images were taken. Thus, UAVDT-DET demonstrates the most pronounced variation in shooting altitudes, while TinyPerson primarily consists of images captured from high-altitude perspectives.

The VisDrone-DET2019 [18] dataset is a well-known aerial dataset captured by drones, developed in a collaboration between the AISKYEYE data mining team and Tianjin University. The dataset contains 6471 images in the training set, 548 images in the validation set and 1610 images in test set. It includes 10 object categories: pedestrian, people, bicycle, car, van, truck, tricycle, awning-tricycle, bus, and motor. This dataset is selected as the primary dataset for our experiments due to its moderate scale, rich category diversity, ability to effectively demonstrate experimental performance, and stable training convergence.

The UAVDT-DET [20] dataset, jointly released by the University of Texas at San Antonio, the University of Chinese Academy of Sciences, and Harbin Institute of Technology, is a large-scale and challenging dataset captured by UAVs in diverse and complex scenarios. It comprises 24,778 and 15,598 images in the training set and validation set, respectively. The categories consist of car, truck, and bus. This dataset serves as a valuable resource for advancing research in UAV-based detection and tracking. Compared to the VisDrone dataset, UAVDT-DET contains a larger total number of images. However, since the frames are approximately 80,000 representative samples extracted from 10 h of raw video, a substantial number of highly similar images exist. This characteristic can, to some extent, affect training effectiveness, leading to instability during the training process. Moreover, training on this dataset requires significant GPU resources.

The TinyPerson [19] dataset, published by the University of Chinese Academy of Sciences in Beijing, is the first benchmark designed for low-resolution person detection. It primarily focuses on individuals that are less than 20 pixels in size within highly complex and varied background conditions, containing two categories: sea person and earth person. The dataset consists of 1610 images in total, with 794 images in the training set and 816 images in the validation set. Compared to VisDrone-DET2019 and UAVDT-DET, TinyPerson features smaller and more densely distributed objects, presenting greater challenges for detecting tiny objects. However, a limitation of this dataset is that some images have extremely low resolution, which may adversely affect model training.

### 4.2. Experimental Environment

The experiments are conducted on a high-performance computing system featuring a 64-core Intel Xeon Gold 6326 CPU (2.90 GHz) (Intel Corporation, Santa Clara, CA, USA) and 10 NVIDIA GeForce RTX 3090 GPUs with 24GB of memory (NVIDIA Corporation, Santa Clara, CA, USA). The software setup includes YOLOv8.3.8 from Ultralytics, with Python 3.8.19 as the programming language. The system runs on Ubuntu 20.04.6 LTS, utilizing a deep learning architecture that combines CUDA 11.7, PyTorch 2.0.0, and torchvision 0.15.1.

### 4.3. Evaluation Metrics

The experiments employ multi-dimensional object detection evaluation metrics: precision (P), recall (R), F1-score (F1), Average Precision (AP), mean Average Precision (mAP), mAP@0.5 (at an Intersection over Union threshold of 0.5), mAP@0.5:0.95 (over the IoU threshold range from 0.5 to 0.95 with a step size of 0.05), and Frames Per Second (FPS). Additionally, model complexity is assessed through the number of parameters, computational cost measured in GFLOPs, and memory footprint.

### 4.4. Training Strategies

Unless otherwise specified, all experiments are evaluated on the validation set of the respective datasets. The model is trained for 200 epochs with a batch size of 15. Key hyperparameters include an IoU threshold of 0.7 and an input image size of 640 × 640 pixels. The optimizer is set to auto, with an early stopping mechanism (patience = 50). Automatic Mixed Precision training is enabled. For data augmentation, the mosaic strategy is employed and deactivated during the final 10 epochs to enhance model robustness. To investigate the effectiveness of individual modules without the influence of pre-trained weights, no pre-trained weights are used. Furthermore, the random seed is fixed to ensure the reproducibility of all experiments.

### 4.5. Comparative Experiments Between YOLO11s and YOLO11s-UAV on Different UAV-Based Datasets

To intuitively demonstrate the performance differences between the improved YOLO11s-UAV model and the baseline YOLO11s, we conduct comparative experiments on the validation and test sets of the VisDrone-DET2019 dataset, as well as the validation sets of the TinyPerson and UAVDT-DET datasets.

Table 2 reports the mAP@0.5 performance of our YOLO11s-UAV model compared to the baseline YOLO11s on the VisDrone-DET2019 validation and test sets. Our model consistently outperforms the baseline across all categories, with more pronounced gains for small objects. On the validation set, mAP@0.5 improves by 7.8%, from 38.2% to 46.0%, while on the completely unseen test set, it rises by 5.9%, from 31.4% to 37.3%. These results confirm the superior feature extraction capability of our model, particularly for small object detection. Moreover, its performance on the test set demonstrates strong generalization to unseen data.

As shown in Table 3, our model also achieves significant mAP@0.5 improvements on the TinyPerson dataset, which comprises low-resolution UAV imagery. The overall mAP increases by 7.2%, with notable gains of 6.7% and 7.6% for the sea person and earth person categories, respectively, indicating strong small object detection under ultra-low resolution. Generalization experiments on the larger UAVDT-DET dataset further validate our model’s robustness, with a 3.0% overall increase in mAP@0.5. Although a slight decrease is observed in the car category, which is likely due to scene redundancy, the model achieves improvements of 4.6% and 6.1% in the truck and bus categories, respectively. These results demonstrate the model’s strong generalization and adaptability across various UAV-based scenarios.

### 4.6. Comparative Experiments of Different Methods

To comprehensively evaluate the effectiveness of our proposed methods, we conduct a series of comparative experiments focusing on three key components: upsampling methods, downsampling modules, and FPN configurations. The detection results under various settings are summarized in Table 4.

We first assess the impact of different upsampling techniques used in CARIFPN. Compared with the default Nearest and Bilinear interpolation, the DySample series [51] introduces only slight increases in model complexity but shows limited improvements in detection performance. In contrast, CARAFE [45] achieves the highest mAP@0.5 at 46.0% and mAP@0.5:0.95 at 28.0%, along with improved recall, despite a modest reduction in FPS due to its higher complexity.

**Figure 7:**
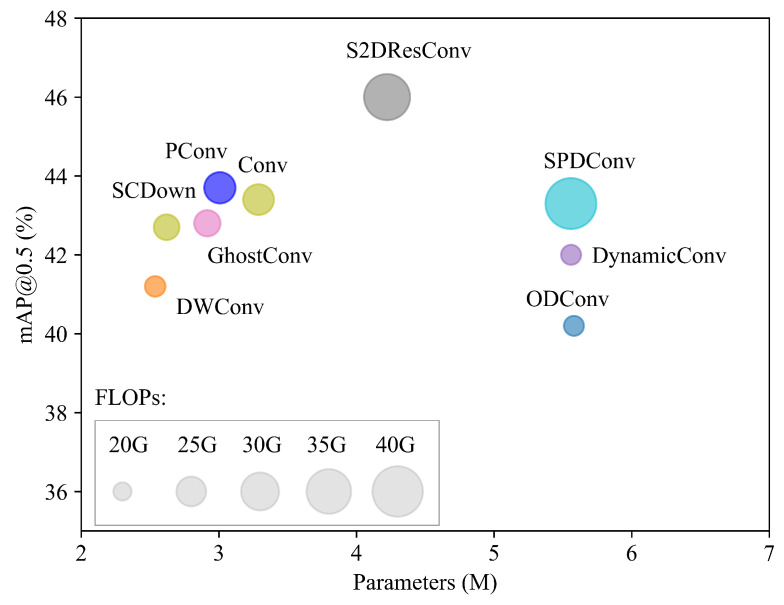
Discrete plot of the impact of different convolutions. The size of the circle represents the relative magnitude of GFLOPs.

We then replace the downsamplers in the YOLO11s-UAV backbone with several representative alternatives, including DWConv [11], ODConv [12], SCDown [29], GhostConv [13], DynamicConv [52], SPDConv [46], PConv [53] and our designed S2DResConv. Figure 7 provides an intuitive comparison among the evaluated downsampling modules in terms of mAP@0.5, the number of parameters, and GFLOPs. Among them, S2DResConv achieves the best detection performance, with a 2.6% increase in mAP@0.5 over the standard Conv module, while maintaining competitive model complexity. This indicates that S2DResConv has advantages in information retention and facilitating the capture of multi-scale contextual information for small objects.

**Figure 8:**
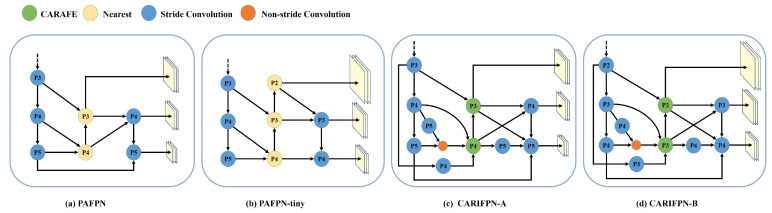
Different designs of the FPN structure, consisting of (**a**) PAFPN, (**b**) PAFPN-tiny, (**c**) CARIFPN-A, and (**d**) CARIFPN-B. The dashed arrows indicate the omission of the previous downsampling feature layers.

Lastly, we evaluate different FPN designs integrated with the original Nearest upsampling or CARAFE. PAFPN-tiny is a lightweight variant of PAFPN tailored for small object detection. It introduces an additional upsampling layer at the P2 level and removes the downsampling path at P5 to better preserve fine-grained spatial features. Compared to CARIFPN-B, CARIFPN-A retains the original five downsampling stages in the backbone, following the design of YOLO11. Figure 8 clearly illustrates the feature fusion pathways of all four FPN structures. Experimental results show that CARIFPN-B achieves the highest mAP@0.5 at 46.0%, representing a 5.7% improvement over the baseline PAFPN, while also reducing parameters by 68% and achieving the best real-time performance among all configurations. These results demonstrate that CARIFPN’s refined design effectively prevents excessive information loss for small objects. This is achieved by reducing the number of downsampling stages. The skip connections in CARIFPN strengthen cross-scale feature fusion. At the same time, CARAFE enriches semantic reconstruction during upsampling.

### 4.7. Comparative Experiments of FlexSimAM

In the baseline, all original C3k2 modules are retained. Meanwhile in the other schemes, we replace all C3k2 modules in the entire model with the proposed FlexSimAM. Since FlexSimAM retains the unique mechanism of c3k, the Boolean value, which can be set to true or false, determines whether we enable FlexSimAM in each layer or revert to the C2f module used in YOLOv8. Based on this, we implement three sets of schemes, with a checkmark indicating that the Boolean value of c3k is set to true, and otherwise, it is false.

The experimental results in Table 5 show that when we replicate the YOLO11 setting for c3k, mAP@0.5 increases by 0.6% in scheme 1. However, when all c3k values are set to true in scheme 2, mAP@0.5 decreases by 0.2% compared to scheme 1. When we keep the c3k setting from scheme 1 for the backbone and set all c3k values in the neck to true, the performance improves to the optimal level. Compared to the baseline, we observe increases of 2.0% in recall, 1.5% in mAP@0.5, and 1.0% in mAP@0.5:0.95, while the number of parameters decreases by 0.18M and GFLOPs decrease by 0.9G in scheme 3.

**Figure 9:**
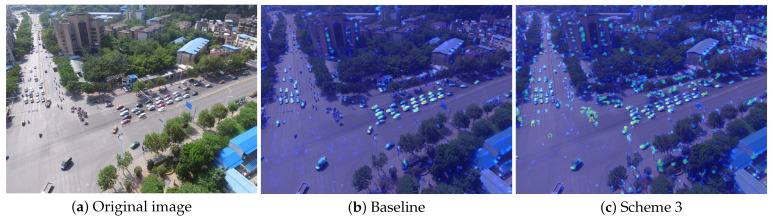
(**a**) Original image and heatmaps generated by Grad-CAM of (**b**) baseline and (**c**) scheme 3.

Additionally, we utilize the Gradient-weighted Class Activation Mapping (Grad-CAM) technique to generate heatmaps in Figure 9. It illustrates that the introduction of the SimAM [48] mechanism in scheme 3 enables our model to expand the scope of its focus on small objects in each image, effectively reducing the probability of missed detection for small objects. These experiments demonstrate that maintaining the original c3k setting in the backbone network helps preserve fundamental local feature extraction capabilities, while fully enabling the attention enhancement module in the neck network, which demands more complex feature fusion, can more effectively strengthen the extraction and integration of subtle features.

### 4.8. Ablation Experiments

**Figure 10:**
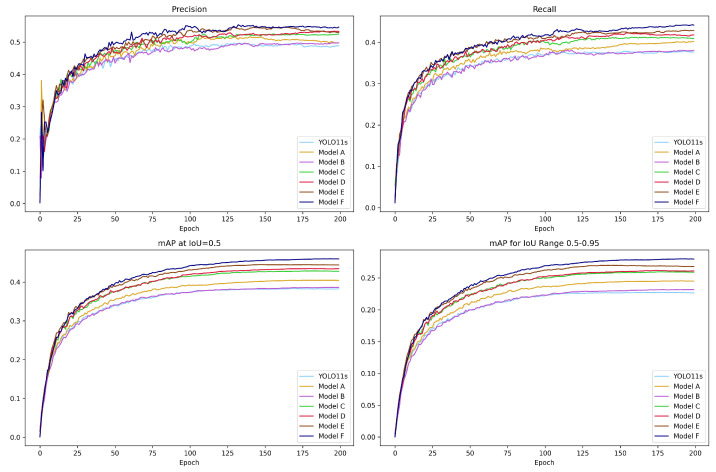
Metric curves of ablation experiments, showing precision, recall, mAP@0.5, and mAP@0.5:0.95 for the baseline model and Models A to F.

To evaluate the individual and combined contributions of our proposed components, we conduct a series of ablation experiments, as summarized in Table 6. Based on the YOLO11s framework, we introduce three key modifications: the CARIFPN structure, the S2DResConv downsampling module, and the lightweight attention mechanism FlexSimAM. A total of seven configurations, namely YOLO11s and Models A to F, are tested. The performance trends in precision, recall, mAP@0.5, and mAP@0.5:0.95 across training epochs are illustrated in Figure 10.

Introducing S2DResConv alone (Model A) yields a notable 2.2% gain in mAP@0.5, with a corresponding increase in model complexity, demonstrating its effectiveness in preserving spatial details during downsampling. FlexSimAM alone (Model B) achieves a modest 0.4% improvement while reducing model size, validating its parameter-free design. Replacing the original FPN with CARIFPN (Model C) results in a significant 4.6% increase in mAP@0.5 and a sharp reduction in parameters, which can be attributed to CARAFE’s context-aware upsampling, the skip connections that promote multi-scale feature fusion efficiency, and the reduced downsampling.

Building on Model C, the addition of FlexSimAM (Model D) further boosts mAP@0.5 by 5.2% and yields the most lightweight model, reducing parameters by approximately 65%, indicating strong complementarity between the enhanced feature fusion pathway and the SimAM attention mechanism. Incorporating S2DResConv into Model C (Model E) improves mAP@0.5 by 1.7%, resulting in a total gain of 6.3% over the baseline. This shows that the new convolutional downsampling features in the backbone network are beneficial for the feature extraction and fusion of the entire CARIFPN.

Finally, combining all enhancements (Model F) achieves the best overall performance: a 6.4% increase in F1-score, 7.8% in mAP@0.5, and 5.3% in mAP@0.5:0.95, with a 55% reduction in parameters. The performance improvement exceeds the sum of individual contributions, demonstrating synergistic effects among the proposed improvements. GFLOPs remain low, and FPS drops only slightly, making the model highly suitable for deployment on UAVs and efficient real-time detection. These results validate the effectiveness of each proposed improvement and their complementarity in enhancing small object detection capabilities.

### 4.9. Comparative Experiments with Other Models

To further validate the effectiveness of our proposed model in detecting small UAV-based objects, we conduct a comprehensive comparison with several state-of-the-art detectors, including both two-stage and one-stage approaches. The two-stage detectors comprise Faster R-CNN [21] and Cascade R-CNN [22], while the one-stage group includes RetinaNet [23], FSAF [24], the YOLO series, and several models specifically tailored for UAV-based small object detection.

As shown in Table 7, traditional two-stage detectors exhibit high computational complexity, with large parameter counts, significant GFLOPs, and memory overhead, yet relatively low detection performance in UAV scenarios. These characteristics limit their applicability on resource-constrained UAV platforms. While one-stage models such as RetinaNet and FSAF are more efficient, they still fall short in balancing accuracy and computational cost.

Our model significantly outperforms the baseline YOLO11s with a 7.8% improvement in mAP@0.5 and shows superior performance over several YOLO variants. Specifically, it surpasses YOLOv5s [25], YOLOv5s-SPD [46], YOLOv6s [30], YOLOv8s [27], YOLOv9s [28], YOLOv10s [29], YOLOv12s [32], YOLOv13s [33], and DAMO-YOLO [43] by 7.6%, 6.0%, 10.0%, 7.4%, 6.5%, 7.8%, 7.8%, 9.9% and 4.4% in mAP@0.5, respectively. Even when compared to larger and more complex models such as YOLOv8x [27] and YOLO11l, our method maintains a performance margin of 0.9% and 1.6% in mAP@0.5.

Additionally, our model achieves competitive results against recent UAV-specific detection methods, including Drone-YOLO (small) [55], DDSC-YOLO [56], PVswins-YOLOv8s [35], MFFCI-YOLOv8 [57], MARFPNet [58], SDMNet [59], LUDY-S [60], VMC-DETR [61] and BPD-YOLOs [37]. In terms of detection accuracy, our model consistently surpasses these methods. It also achieves this with only 4.2M parameters and a memory footprint of just 8.5MB, both of which are lower than those of the compared models. Furthermore, its computational cost remains acceptable. It is noteworthy that our model matches the detection accuracy of VMC-DETR while using only 36.1 GFLOPs. In contrast, VMC-DETR requires 70.5 GFLOPs, nearly doubling our computational cost. These results demonstrate that our model offers a favorable trade-off between detection performance and model complexity.

In summary, our model achieves high performance while maintaining a lightweight structure suitable for real-time deployment on embedded UAV systems. Its strong performance and efficiency make it well-suited for practical applications in UAV-based areas.

### 4.10. Visualization Analysis

We conduct a qualitative comparison of detection results using the best-performing models trained on the VisDrone-DET2019 dataset. Bounding boxes are visualized at an IoU threshold of 0.45 for (b) YOLOv8s, (c) YOLO11s, and (d) YOLO11s-UAV, compared against ground truth annotations. The selection of YOLOv8s and YOLO11s as visual comparisons is attributed to their status as mainstream general-purpose detectors from the Ultralytics framework. The error analysis is color-coded as follows:

- **Green (True Positives, TP)**: Correct detections where the predicted bounding boxes align accurately with both the position and category of the ground truth.
- **Blue (False Positives, FP)**: Incorrect predictions where non-target regions are falsely identified as objects.
- **Red (False Negatives, FN)**: Missed detections where actual objects are not identified by the model.

**Figure 11:**
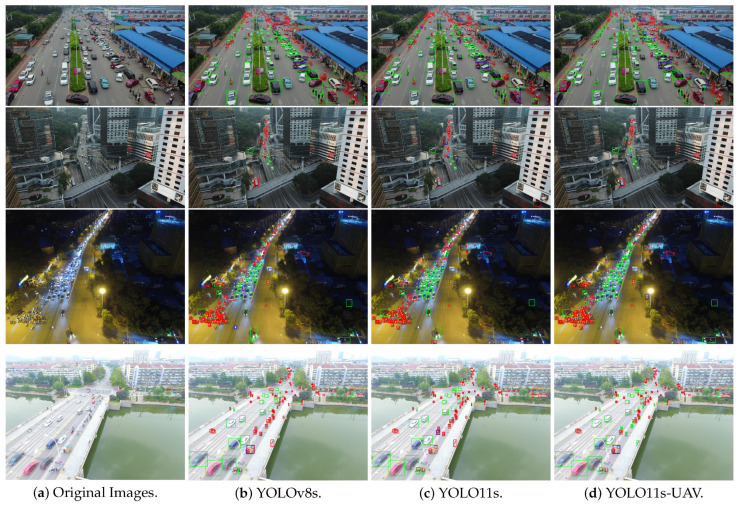
Error breakdown visualization of the representative images in the test set of VisDrone-DET2019. From top to bottom: urban streets, tall buildings, night-time streets, and overexposed river-crossing bridge. The bounding boxes of green, blue and red represent the TP, FP and FN, respectively. From left to right: (**a**) original images, (**b**) YOLOv8s results, (**c**) YOLO11s results, and (**d**) YOLO11s-UAV results.

Representative samples from the unseen test set of the VisDrone-DET2019 dataset are illustrated in Figure 11, covering a range of complex scenes including (from top to bottom): urban streets, high-rise buildings, night-time roads, and overexposed river bridges. The visual comparisons reveal that our proposed YOLO11s-UAV model detects smaller and more distant objects more effectively than YOLOv8s and YOLO11s models. Moreover, it maintains robust detection performance under challenging scenarios such as low illumination and overexposure. These results underscore the superior capability of our model in capturing discriminative features from small objects, particularly in visually complex UAV-based environments.

### 4.11. Hardware Deployment on NVIDIA Jetson

To validate the practical applicability of our YOLO11s-UAV model in real-world UAV scenarios, we deploy it on a resource-constrained embedded platform—the NVIDIA Jetson Orin NX SUPER 8GB hardware module—to measure its practical efficiency metrics. For inference, the models are loaded in their native PyTorch format with a fixed input resolution of 640 × 480 pixels and a batch size of 1. All evaluations are performed within the pre-configured Ultralytics environment running on the factory-imaged Jetson system, utilizing an external camera for live object detection.

Table 8 summarizes the average deployment results on the Jetson Orin NX SUPER platform. YOLO11s achieves a processing speed of 42 FPS, while YOLO11s-UAV operates at 33 FPS, which is 21.4% lower. A significant advantage of the YOLO11s-UAV model is its substantially lower memory consumption of 8.5 MB, representing a 53.6% reduction compared to the 18.3 MB required by YOLO11s. This efficiency is particularly valuable for UAV applications where onboard memory resources are often limited. Power consumption measurements show that YOLO11s-UAV requires 7.3 W during inference, which is 9.0% higher than the 6.7 W consumed by YOLO11s. Notably, YOLO11s-UAV maintains a lower operating temperature of 57.03 °C compared to 58.97 °C for YOLO11s, suggesting improved thermal efficiency despite the higher power draw. This thermal characteristic may contribute to more stable long-term operation in constrained UAV environments.

## 5. Conclusions

In this study, we propose a novel algorithm for UAV-based small object detection, named YOLO11s-UAV, based on the YOLO11s architecture. We design a new Feature Pyramid Network, CARIFPN, to replace PAFPN, which significantly reduces the number of model parameters while enhancing the multi-scale feature extraction and fusion capabilities for small objects. Additionally, we introduce the S2DResConv module into the backbone, which not only mitigates the information loss caused by traditional convolutions but also promotes multi-scale context capture. Furthermore, we design FlexSimAM to replace all C3k2 modules throughout the network. This approach not only results in a more lightweight network structure but also enhances the model’s focus on small object features in complex environments, thereby improving detection accuracy.

Experimental results validate the effectiveness of the combined modules. Our model demonstrates exceptional performance on UAV datasets. Specifically, on the VisDrone-DET2019 aerial imagery dataset, our model achieves a 7.8% improvement in mAP@0.5 on the validation set (reaching 46.0%) and a 5.9% improvement on the test set (increasing from 31.4% to 37.3%) compared to the baseline YOLO11s. Similarly, on the TinyPerson dataset, our model shows a 7.2% improvement, and on the UAVDT-DET dataset, it yields a 3.0% increase in mAP@0.5. Furthermore, deployment tests on the NVIDIA Jetson Orin NX SUPER platform validate its practical efficiency, achieving a real-time speed of 33 FPS with 53.6% lower memory usage than the baseline, confirming its suitability for resource-constrained UAV platforms.

Our model not only demonstrates significant potential for practical applications through its technical innovations, but also confronts the ongoing challenges in small object detection. While our model outperforms existing approaches, the overall detection accuracy remains below the desired threshold, and its robustness under extreme weather conditions (e.g., heavy snow, dense fog, and sandstorms) requires further validation due to the current lack of corresponding datasets. Moving forward, we plan to improve loss functions tailored for small object detection to achieve enhanced accuracy without increasing computational cost. Concurrently, we will investigate lightweight network pruning and quantization techniques to further optimize inference speed. In the long term, we aim to collect or collaborate in building specialized datasets covering diverse adverse weather and environmental conditions, thereby comprehensively improving the model’s generalization and robustness for real-world UAV deployments. Through these efforts, we strive to achieve a balanced advancement in accuracy, speed, and resource efficiency for embedded UAV systems. 

## Figures and Tables

**Table 1:** Detection results of improved model with different specifications of YOLO11 on the VisDrone-DET2019 dataset (metrics: P, precision; R, recall; mAP, mean Average Precision; Param(M), parameter count in millions; GFLOPs, Giga Floating-point Operations Per Second; FPS, Frames Per Second).

Models	P(%)	R(%)	mAP@0.5(%)	mAP@0.5:0.95(%)	Param(M)	GFLOPs	FPS
YOLO11n	42.1	33.1	32.0	18.5	2.6	6.3	330
YOLO11s	48.7	37.6	38.2	22.7	9.4	21.3	192
YOLO11m	54.1	41.5	42.7	26.1	20.0	67.7	144
YOLO11l	56.2	42.5	44.4	27.4	25.3	86.6	98
YOLO11x	57.9	44.5	46.1	28.6	56.8	194.5	38

**Table 2:** Comparison of mAP@0.5 of improved model with baseline YOLO11s on the VisDrone-DET2019 dataset (the increase is indicated in blue text).

Set	Models	Pedestrian	People	Bicycle	Car	Van	Truck	Tricycle	Awning	Bus	Motor	All
Val	YOLO11s	40.6	31.4	11.7	79.2	44.8	35.1	25.9	15.9	55.3	42.2	38.2
	Ours	52.4	42.3	18.1	84.9	50.8	40.7	34.7	19.9	61.7	54.1	46.0(+7.8)
Test	YOLO11s	26.0	13.5	8.3	70.6	37.0	37.9	16.3	19.7	56.1	28.3	31.4
	Ours	34.6	21.9	12.6	77.8	42.6	42.7	20.6	23.5	59.9	36.3	37.3(+5.9)

**Table 3:** Detection results on validation set of different UAV-based datasets (the increase for each category is indicated in blue text).

Datasets	Images	Instances	Models	Categories	P(%)	R(%)	mAP@0.5(%)	mAP@0.5:0.95(%)
TinyPerson [19]	786	18,508	YOLO11s	All	34.8	18.5	16.0	5.7
				Sea person	38.2	21.2	18.1	7.0
				Earth person	31.3	15.8	13.9	4.5
			Ours	All	38.7	25.4	23.2(+7.2)	8.3
				Sea person	41.7	27.4	24.8(+6.7)	9.4
				Earth person	35.7	23.5	21.5(+7.6)	7.2
UAVDT-DET [20]	15,598	307,923	YOLO11s	All	42.8	34.4	34.6	21.3
				Car	72.6	71.6	76.0	45.6
				Truck	37.8	9.5	14.1	9.2
				Bus	17.8	22.2	13.7	9.2
			Ours	All	47.9	35.1	37.6(+3.0)	23.7
				Car	78.9	64.2	74.4	45.5
				Truck	38.3	13.9	18.7(+4.6)	11.8
				Bus	26.6	27.0	19.8(+6.1)	13.8

**Table 4:** Detection results of the application of different methods to our model on the VisDrone-DET2019 dataset (The best results are marked in bold).

Methods	Precision(%)	Recall(%)	mAP@0.5(%)	mAP@0.5:0.95(%)	Param(M)	GFLOPs	FPS
Nearest	**55.3**	43.2	45.2	27.3	**4.090**	**35.0**	**169**
Bilinear	52.8	43.5	44.7	26.9	4.091	35.1	97
DySample [51]	54.4	43.1	45.2	27.5	4.099	35.1	138
DySample+ [51]	54.9	43.8	45.4	27.6	4.107	35.1	128
DySample-S [51]	53.6	43.7	45.1	27.5	4.091	**35.0**	123
DySample-S+ [51]	55.0	42.9	45.1	27.5	4.092	**35.0**	129
CARAFE [45]	54.4	**44.2**	**46.0**	**28.0**	4.223	36.1	118
Conv	52.8	41.9	43.4	26.2	3.289	25.5	**161**
DWConv [11]	50.4	39.8	41.2	24.4	**2.538**	20.8	56
ODConv [12]	48.9	39.2	40.2	23.9	5.581	**20.6**	97
SCDown [29]	52.7	40.8	42.7	25.8	2.621	22.9	54
GhostConv [13]	52.3	40.8	42.8	25.7	2.917	23.2	118
DynamicConv [52]	50.9	40.3	42.0	24.9	5.560	**20.6**	125
SPDConv [46]	53.3	41.7	43.3	26.1	5.559	40.3	147
PConv [53]	52.6	42.3	43.7	26.5	3.008	25.8	116
S2DResConv	**54.4**	**44.2**	**46.0**	**28.0**	4.223	36.1	118
PAFPN [9]	50.5	40.0	40.3	24.2	13.007	**33.5**	105
PAFPN-tiny	53.1	41.9	43.6	26.4	10.956	38.8	70
CARIFPN-A	51.1	40.3	40.5	24.4	13.416	34.6	86
CARIFPN-B	**54.4**	**44.2**	**46.0**	**28.0**	**4.223**	36.1	**118**

**Table 5:** Detection results of different schemes for FlexSimAM (The best results are marked in bold; A checkmark (✓) indicates that the Boolean value of c3k is set to true, and a cross mark (×) indicates false).

Parameter c3k	Baseline	Scheme 1	Scheme 2	Scheme 3
Layer 2	×		✓	
Layer 4	×		✓	
Layer 6	×	✓	✓	✓
Layer 12	×		✓	✓
Layer 16	×		✓	✓
Layer 19	×		✓	✓
Layer 22	×		✓	✓
Layer 26	×	✓	✓	✓
Precision(%)	54.4	52.5	**55.3**	54.4
Recall(%)	42.2	**44.2**	42.5	**44.2**
mAP@0.5(%)	44.5	45.1	44.9	**46.0**
mAP@0.5:0.95(%)	27.0	27.3	27.2	**28.0**
Param($10^{6}$)	4.403	4.256	**4.210**	4.223
GFLOPs	37.0	36.5	**35.8**	36.1
FPS	101	99	103	**118**

**Table 6:** Detection results of ablation experiments on the VisDrone-DET2019 dataset (The increase and decrease are indicated in blue and red text respectively and the best results are marked in bold; A checkmark (✓) indicates that the component is included in the model).

Models	CARIFPN	FlexSimAM	S2DResConv	F1(%)	mAP@0.5(%)	mAP@0.5:0.95(%)	Param(M)	GFLOPs	FPS
YOLO11s				42.4	38.2	22.7	9.4	21.3	**192**
Model A			✓	44.5	40.4(+2.2)	24.5	13.8	34.6	116
Model B		✓		43.4	38.6(+0.4)	23.2	8.7(−0.7)	**20.2**	128
Model C	✓			46.0	42.8(+4.6)	26.0	3.5(−5.9)	26.4	167
Model D	✓	✓		46.7	43.4(+5.2)	26.2	**3.3** (−6.1)	25.5	161
Model E	✓		✓	47.5	44.5(+6.3)	27.0	4.4(−5.0)	37.0	101
Model F	✓	✓	✓	**48.8**	**46.0** (+7.8)	**28.0**	4.2(−5.2)	36.1	118

**Table 7:** Detection results of comparative experiments on the VisDrone-DET2019 dataset (The model citation in the table refers to data directly from the referenced paper and the best results are marked in bold).

Models	Year	mAP@0.5(%)	mAP@0.5:0.95(%)	Param(M)	Memory(MB)	GFLOPs	FPS
Faster R-CNN-R50	2015	33.5	18.8	41.4	161.3	208	37
RetinaNet-R18	2017	21.6	11.8	21.1	86.0	190	25
Cascade R-CNN-R50	2018	33.2	19.5	69.4	267.9	236	86
FSAF-R50	2019	30.7	16.4	36.4	145.4	207	27
YOLOv5s	2020	38.4	22.9	9.1	17.7	23.8	71
YOLOv5s-SPD	2022	40.0	24.0	10.7	20.6	41.4	33
YOLOv6s	2022	36.0	21.5	16.3	31.3	44.0	76
DAMO-YOLO [54]	2022	41.6	24.6	-	-	-	-
YOLOv8s	2023	38.6	23.1	11.1	21.5	28.5	115
YOLOv8x	2023	45.1	27.9	68.1	130.4	257.4	62
Drone-YOLO (small) [55]	2023	44.3	27.0	10.9	-	-	-
YOLOv9s	2024	39.5	23.5	7.1	14.5	26.7	23
YOLOv10s	2024	38.2	22.9	8.0	15.8	24.5	**247**
YOLO11s	2024	38.2	22.7	9.4	18.3	**21.3**	192
YOLO11l	2024	44.4	27.4	25.3	48.8	86.6	98
DDSC-YOLO [56]	2024	42.2	25.5	5.0	-	-	-
PVswin-YOLOv8s [35]	2024	43.3	26.4	-	21.6	-	161
MFFCI-YOLOv8 [57]	2024	40.6	-	10.3	-	25.1	154
MARFPNet [58]	2024	44.3	27.1	-	13.0	40.9	-
SDMNet [59]	2024	43.8	25.3	93.2	178	378.3	-
YOLOv12s	2025	38.2	22.8	9.3	18.1	21.4	78
YOLOv13s	2025	36.1	21.2	9.0	17.8	20.7	95
LUDY-S [60]	2025	41.7	-	10.3	20.5	-	194
VMC-DETR [61]	2025	45.9	27.9	-	-	70.5	-
BPD-YOLOs [62]	2025	45.0	27.4	5.8	-	36.7	-
YOLO11s-UAV (Ours)	2025	**46.0**	**28.0**	**4.2**	**8.5**	36.1	118

**Table 8:** Deployment performance comparison on Jetson Orin NX SUPER (Note that temperature refers to GPU core temperature; The best results are marked in bold).

Model	FPS	Memory (MB)	Power (W)	Latency (ms)	Temperature (°C)
YOLO11s	**42**	18.3	**6.7**	**23.6**	58.97
YOLO11s-UAV	33	**8.5**	7.3	30.5	**57.03**

## Data Availability

The data presented in this study are available in the following public repositories: the VisDrone-DET2019 dataset is available at https://github.com/VisDrone (accessed on 30 January 2026); the UAVDT-DET dataset is available at https://opendatalab.org.cn/OpenDataLab/UAVDT (accessed on 30 January 2026); the TinyPerson dataset is available at https://github.com/ucas-vg/PointTinyBenchmark (accessed on 30 January 2026).

## Abbreviations

The following crucial abbreviations are used in this manuscript:

YOLO	You Only Look Once
UAVs	Unmanned Aerial Vehicles
FPN	Feature Pyramid Network
CARIFPN	Content-Aware Reassembly and Interaction Feature Pyramid Network
CARAFE	Content-Aware ReAssembly of FEature
RepGFPN	Reparameterized Generalized-FPN
S2DResConv	Space-to-Depth for Dilation-wise Residual Convolution
SPD	Space-to-Depth
DWR	Dilation-wise Residual Convolution
RR	Regional Residualization
SR	Semantic Residualization
SimAM	Simple, Parameter-Free Attention Module
FlexSimAM	Flexible SimAM
C2f	Channel to Feature
SPPF	Spatial Pyramid Pooling—Fast
PSA	Pointwise Spatial Attention
DSConv	Depthwise Separable Convolution
P	Precision
R	Recall
mAP	mean Average Precision
F1	F1-Score
Param	Parameters
GFLOPs	Giga Floating-point Operations Per Second
FPS	Frames Per Second
